# Filament-Filament Switching Can Be Regulated by Separation Between Filaments Together with Cargo Motor Number

**DOI:** 10.1371/journal.pone.0054298

**Published:** 2013-02-14

**Authors:** Robert P. Erickson, Steven P. Gross, Clare C. Yu

**Affiliations:** 1 Department of Physics and Astronomy, University of California Irvine, Irvine, California, United States of America; 2 Department of Developmental and Cell Biology, University of California Irvine, Irvine, California, United States of America; University of Cambridge, United Kingdom

## Abstract

How intracellular transport controls the probability that cargos switch at intersections between filaments is not well understood. In one hypothesis some motors on the cargo attach to one filament while others attach to the intersecting filament, and the ensuing tug-of-war determines which filament is chosen. We investigate this hypothesis using 3D computer simulations, and discover that switching at intersections increases with the number of motors on the cargo, but is not strongly dependent on motor number when the filaments touch. Thus, simply controlling the number of active motors on the cargo cannot account for *in vivo* observations that found reduced switching with increasing motor number, suggesting additional mechanisms of regulation. We use simulations to show that one possible way to regulate switching is by simultaneously adjusting the separation between planes containing the crossing filaments and the total number of active motors on the cargo. Heretofore, the effect of filament-filament separation on switching has been unexplored. We find that the switching probability decreases with increasing filament separation. This effect is particularly strong for cargos with only a modest number of motors. As the filament separation increases past the maximum head-to-head distance of the motor, individual motors walking along a filament will be unable to reach the intersecting filament. Thus, any switching requires that other motors on the cargo attach to the intersecting filament and haul the cargo along it, while motor(s) engaged on the original filament detach. Further, if the filament separation is large enough, the cargo can have difficulty proceeding along the initial filament because the engaged motors can walk underneath the intersecting filament, but the cargo itself cannot fit between the filaments. Thus, the cargo either detaches entirely from the original filament, or must dip to the side of the initial filament and then pass below the crossing filament.

## Introduction

An integral part of intracellular transport involves a cargo, hauled along a filament by molecular motors, switching onto another filament at filament intersections. How such switching occurs is not well understood, though it is known that a single engaged Myosin-V motor can switch onto another actin filament at an intersection [1]. (We will refer to ‘engaged’ motors as motors that are walking along the filament and hauling the cargo.) In addition, a popular scenario for switching is the tug-of-war hypothesis [2]–[5] in which, as a cargo approaches an intersection between 2 filaments, some of the inactive motors on the cargo can attach to the nearby filament, and then a tug-of-war ensues between the motors on the two filaments. The outcome of the tug-of-war determines which filament is ultimately used to transport the cargo.

Past studies [2] show that cells can regulate transport in part by changing the probability that a cargo switches at actin-actin intersections. For example, pigment granules (melanosomes) in *Xenopus* melanophore cells manipulated to have only actin filaments and no microtubules have almost no probability (0% to 6%) of switching at actin-actin intersections when the melanosomes are trying to spread out away from the nucleus (dispersion), but have a 50% chance of switching at intersections when they have been given the signal to aggregate toward the middle of the cell [2]. This may be correlated with the number of motors on the cargo: biochemistry indicates that there are about 90 Myosin-V motors per granule during dispersion but only 60 motors per melanosome during aggregation [6], though in principle many of these motors could be inactive, so this merely puts an upper bound on the number of active motors potentially present on the cargo. By ‘active motors’, we mean motors that can in principle attach to a filament and walk along it, though they may not be attached to a filament if, for example, they cannot reach the filament. In contrast, inactive motors cannot attach to or walk along the filament. Because the switching probability decreased as the number of cargo-bound Myosin-V motors increased [6], we had previously hypothesized that motor number might regulate filament switching dynamics via a tug-of-war mechanism. We reasoned that the more motors there were on the cargo, the more motors that would be actively hauling the cargo along a filament and hence, the harder it would be for another motor on the cargo to pull the cargo onto an intersecting filament because it would have to overcome several motors walking along the original filament.

Here, within the context of a three-dimensional model, we investigated this possibility using computer simulations. We found that as the number of motors on the cargo increased, the switching probability increased slightly, contrary to *in vivo* experiments and our initial expectation. Thus, merely changing the number of motors on the cargo could not account for the much larger change in switching probability that we observed experimentally [2]. Instead, our simulations suggest that there must exist other molecular mechanisms, contributing the majority of the effects.

We therefore investigated whether one way to control switching might be to adjust the number of active motors on the cargo together with the separation normal to the parallel planes containing the filaments. (We will refer to this separation as the vertical separation between the filaments.) If filaments are touching, then motors can either step over or switch to the intersecting filament. In our simulations, we incorporated the ability of *single* myosin-V to directly switch between crossed filaments [1]; obviously this ability must disappear as filament-filament spacing increases and the motor cannot reach the second filament. We incorporated this into our model by having a switching probability that started at the experimentally measured value of 50% when filaments were touching and linearly decreased to zero when the filaments were spaced at 80 nm (since the measured distance between the motor heads is 74 nm [7]). Thus, as the vertical separation between filaments increased, the actively engaged motors had difficulty switching filaments. However, previously unengaged motors on the cargo could still attach to the intersecting filament, so at large filament separations, the greater the number of total motors the cargo had, the more easily it could switch. In addition, a second factor also comes into play: even though the motors could walk underneath the intersecting filament, the cargo could not fit between the filaments, and so the cargo either detached, or dipped underneath the crossing filament and along side the initial filament, or additional motors helped the cargo to climb over the crossing filament and continue on its way along the original filament. We found that these two effects contributed in interesting ways to switching. Nevertheless, the fact that the switching probability increased as the total number of motors on the cargo increased, contrary to experiment [8], indicates that the other mechanisms must be involved in regulating cargo switching.

## Methods

### Monte Carlo Simulations

To theoretically address these questions, we performed a fully three-dimensional Monte Carlo simulation [8]. Generally speaking, Monte Carlo is an approach to computer simulations in which an event A occurs with a certain probability P_A_ where 0 ≤ P_A_ ≤ 1. In practice, during each time step, a random number *x* is generated with uniform probability between 0 and 1. If *x* ≤ P_A_, event *A* occurs; if *x* > P_A_, event *A* does not occur.

Our simulations were carried out as follows [8]. We started with a three dimensional spherical cargo with a diameter of 0.5 microns. The cargo was subjected to rotational and translational diffusion according to the equations presented below and in the supplement S1. To this cargo, we attached myosin motors at random points on the surface of the cargo because this is likely the way myosin motors are arranged on cargos [2], [8], [9]. Each motor is modeled as a straight rod 60 nm in length [10], which acts as a spring (of spring constant 0.32 pN/nm) when stretched but has no restoring force when compressed. The values of the simulation parameters for the motor are given in Table S1. The motor could pivot freely about the point of attachment to the cargo surface, but it was not allowed to go into or under the cargo surface. The motor was not subject to bending or torsion. In our simulations all the motors on the cargo were active, i.e., they could potentially attach to the filament and walk along it if the filament was within reach. However, all the motors not necessarily were engaged in hauling the cargo. We started the simulation so that potentially one or more motors could bind to an actin filament (7 nm diameter). The motors then moved the cargo along the actin, taking 36 nm steps. While the technical details of the simulation are in the supplement S1 along with the parameter values (see Table S2), the general idea is that at each time step *Δt*, we consider all motors present, calculate all forces acting upon them, and then ask what each of them does.

**Figure 1:**
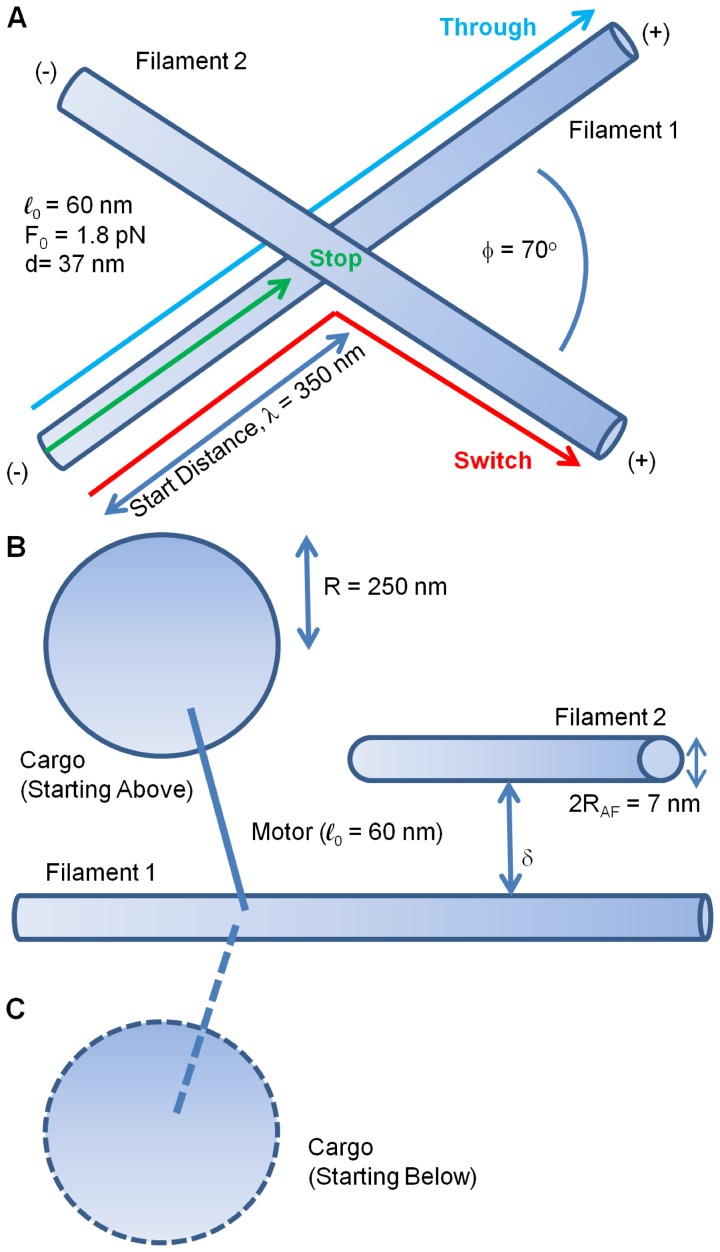
Cargo filament geometry used in simulations. (A) Geometry of two actin filaments crossing. (B) Cargo initially starts above filament 1. (C) Cargo initially starts below filament 1.

The key issue is what occurs when the cargo approaches an intersection (Figure 1A). There are 2 possible processes involved in a cargo switching from one filament to another. First, an engaged motor hauling the cargo along the first filament switches onto the intersecting filament [1]. During the switch, we envision one head of the motor on the first filament and the second head of the motor attaching to the second filament. Second, an unengaged ‘passenger’ motor on the cargo attaches to the intersecting filament and tries to walk along the new filament.

In our simulation two actin filaments intersect at an angle of 70 degrees that is the angle that an actin filament makes with a branching actin filament due to Arp2/3. The results for 70 degrees should be qualitatively the same for other angles between intersecting filaments. (Any angle of intersction is possible for actin filaments that inhabit different planes).

We did not allow the myosin motor to change its basic azimuthal (rotational) position on the actin filament. There were in principle several scenarios. In the first, the motor walked along a filament, and came to a second filament lying on top of and across the first, blocking its path (Figure 1B). Suppose the vertical separation was 0 nm. By vertical separation, we mean the separation normal to the parallel planes containing the filaments. To continue on the filament it started on, a motor had to step over this second filament. It could also switch to the intersecting filament as has been found experimentally [1]. We set the switching rate to 19/sec in this case in order to be consistent with the experimental switching probability of 48% for a single myosin motor [1]. We varied the vertical separation from 0 to 80 nm. 80 nm is just beyond 74 nm, the maximum distance between the two heads of a myosin motor [7]. We linearly decreased the switching rate of a single engaged motor as the vertical separation increased to account for the increased difficulty of a motor walking along the first filament to reach the intersecting filament. The rate went to zero for a vertical separation of 80 nm. This can be described by the formula: Γ = (19/sec) [1-(d_f_/80 nm)] where Γ is the rate at which an engaged motor switches filaments and d_f_ ≤ 80 nm is the vertical separation between filaments. For d_f_ > 80 nm, Γ  =  0.

Switching also occurred when other (previously unengaged) motors on the cargo attached to the intersecting filament, and started to walk along it, forcing the motors on the original filament to detach. As the vertical separation initially increased, motors walking along the first filament were no longer able to step over the second filament, so they either detached or else they switched to the second filament. As the vertical separation between the filaments increased, motors detached at or before the intersection, or switched to the crossing filament, or they walked along the initial filament and went underneath the crossing filament. However, in this last case, the cargo was too big to go between the filaments, so the motors and cargo either detached from the initial filament, or the cargo, which underwent Brownian motion, dipped to the side of the intersection and underneath the crossing filament. If motors attached to the crossing filament, the cargo could switch and move along this filament. When motors attached to the obstructing filament, the result was a tug-of-war between the motors on the original filament and those on the new filament. The more motors that there were on the cargo, the greater the switching probability.

Another possibility was that the motor started out on the other side (bottom) of the filament (Figure 1C), so that it could walk unimpeded along the initial filament, and was expected to frequently not ‘see’ the other filament. In the following we simulated the cases with the motor starting along the top or the bottom of the filament independently.

We start by describing how we simulated transport of a cargo with motors attached. Our basic algorithm is as follows. Consider one or more motors attached at random points to the cargo surface. The cargo was then suspended above the actin filament (AF), with a well-defined separation distance between the bottom of the cargo and the top of the actin, and the motors were each given an opportunity to attach to the actin. Eventually, a motor attached to the filament, and because we assumed saturating ATP, the cargo began to advance. As the cargo traveled along the AF, at each time step of the simulation, each motor on the cargo was given the opportunity to detach from the AF if it was attached, or to attach if it was detached (and geometrically could reach the AF). If a motor was attached to an AF, then there was some probability that it would bind and hydrolyze ATP, and subsequently take a step. Although Myosin-V is a two-headed motor, we modeled each motor by a single myosin head that hydrolyzed ATP in such a way that Michaelis-Menten kinetics was obeyed. The probabilities of a motor detaching from the AF, releasing ATP, and taking a step all depended on the load on the motor (see supplement S1). Because the cargo exerted force on the engaged motors, this load on a motor had contributions from the force externally applied to the cargo, from the other motors that were pulling the cargo, and from thermal fluctuations. If ATP was bound to the motor head, then the probability of detachment increased exponentially with load if the load was less than the stalling force of the motor. If no ATP was bound to the head and the load F was less than the stalling force F_0_, then the detachment probability was proportional to the probability of taking a step which decreased with increasing load as [1-(F/F_0_)^2^]. If the load was greater than the stalling force, then there was a constant rate of detachment. The thermal fluctuations randomly rotated and translated the cargo that, in turn, could stretch the motor linkage and exert a load on the motor. (See below for further details on thermal fluctuations.) Once all the motors had been given a chance to step, the cargo was translated and rotated according to the force and torque to which it was subjected.

The cargo traveled along the actin filament until it either fell off before or at the filament-filament intersection (“stopped”), moved through the intersection along the initial filament and fell off later (“passed through”), or switched and ended up moving along the second filament (Figure 1) before detaching from the second filament (“switched”). The vertical separation between the filaments was fixed between 0 and 80 nm. As a cargo approached an intersection, idle motors that were not attached to the initial filament A could attach to the intersecting filament B. In addition, motors initially attached to filament A could switch to filament B [1] with a rate that decreased linearly from 19/sec for filaments that were touching to 0/sec for filaments that were beyond the motors' reach at 80 nm apart. At intersections, if one group of motors was attached to filament A and, at the same time, another group to filament B, there was a ‘tug-of-war’ between the two groups, and the outcome of the tug-of-war was decided by whether one group of motors completely detached, allowing the other group to transport the cargo along the filament. Alternatively, cargos could just get stuck at intersections if the conflict was not resolved. By “stuck”, we mean that the cargo detached from the actin filament at or before the intersection. In most cases where the cargo was stuck, it stopped at the intersection until it finally detached. Another way in which a cargo could get stuck at an intersection was when there was enough vertical separation to allow motors, but not the cargo, to pass between the filaments. In other words, a motor was thin enough to pass underneath the crossing filament even though the cargo that it was carrying could not. Note that myosin motors are about 60 nm long [10] while actin filaments are 7 nm wide, so that the motor heads could be past the crossing filament while the cargo is stuck on the other side of the filament. This case was classified as ‘cargo stopped’. If thermal fluctuations pushed the cargo to the side, then it could dip under the crossing filament and pass through the intersection. The probability of this happening increased with increasing vertical separation between filaments.

To obtain a standard error of outcome, i.e., “stopped,” “passed through,” or “switched,” of no greater than approximately 0.3% probability, we simulated 25,000 independent scenarios for each data point shown in the plots. A derivation of the estimate of standard error is given in the supplement S1. Each simulation scenario, or run, corresponded to its own unique random attachment of motors over the entire spherical surface of the cargo.

In our simulations, the spherical cargo was subjected to thermal fluctuations that we divided into translational and rotational components. The equation of the cargo's translational motion is given by the Langevin equation:
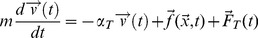
(1.1)where *m* is the cargo's mass and 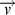
is the cargo's velocity. The drag force on the cargo is proportional to its velocity with the drag coefficient 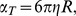 where *R* is the cargo's radius and 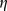 is the coefficient of viscosity that is the kinematic viscosity multiplied by the specific weight of the fluid. 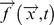 is the sum of the forces on the cargo due to an external force of magnitude F_L_ and the force of the engaged motors pulling on the cargo. One can solve this equation for the position of the cargo at time step *t+*Δ*t* [8]:
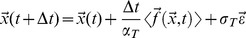
(1.2)where 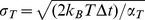 is the standard deviation of a normal distribution and 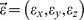 is a vector in Cartesian coordinates of the laboratory frame of reference that represents three independent random variates drawn on a normal distribution having zero mean and unit standard deviation.

For the cargo's rotational motion, the corresponding Langevin equation is
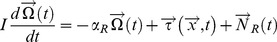
(1.3)where 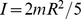 is the moment of inertia of a solid spherical cargo, and 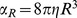
is the drag coefficient proportional to the angular velocity 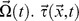 is the torque on the cargo referenced from the center of mass due to the engaged motors. 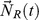 is the rapidly varying random torque due to the thermal fluctuations of the environment. One can solve this equation for the change in orientation of the cargo at each time step [8]. The formulas are analogous to Eq. (1.2).

We assume that the actin filaments are not subject to thermal fluctuations because their persistence length of 9 μm [11] is much longer than the 300 nm distance between actin intersections [2]. Roughly speaking, the persistence length is the distance over which the filament is straight before bending. The actin will be quite stiff between intersecting filaments because the crossing filaments help to hold the actin filament in place, and therefore the thermal fluctuations of the actin filaments will be negligible.

## Results

### The total number of motors on the cargo had a modest effect on filament switching for a small vertical separation between filaments

**Figure 2:**
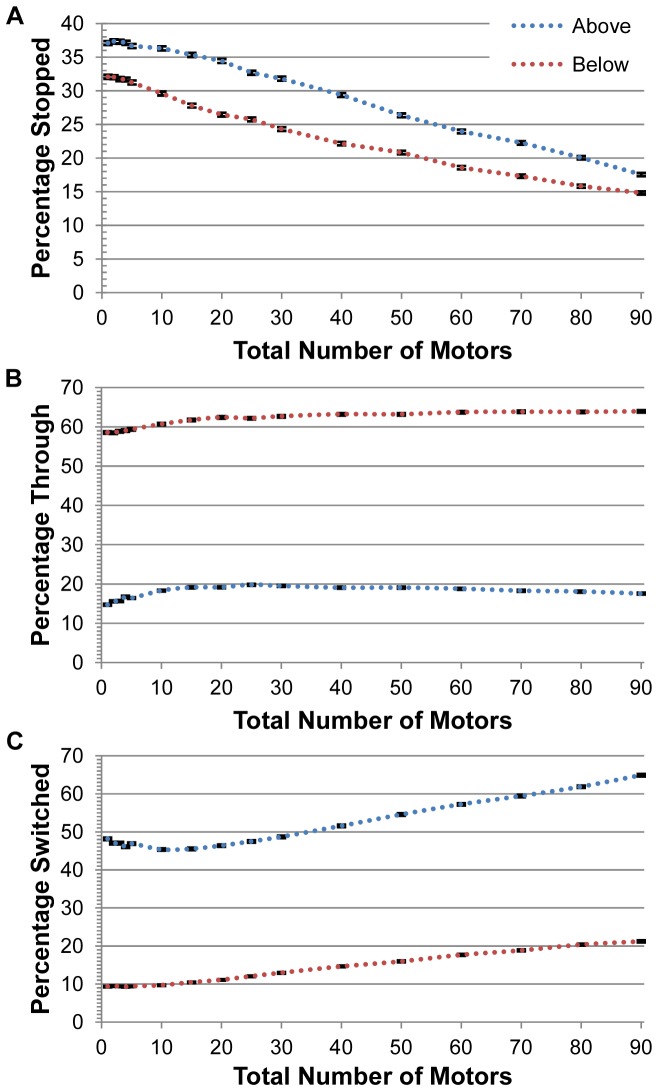
Probability of different outcomes for a cargo approaching an intersection versus the total number of motors on the cargo with no vertical separation between filaments. “Above” means the cargo started on top of the initial filament. “Below” means the cargo started on the bottom of the initial filament. The intersecting filament lay on top of the initial filament at an angle of 70 degrees. (A) Probability that a cargo got stopped at an intersection, i.e., probability that a cargo detached at an intersection or before reaching the intersection. (B) Probability that a cargo went through an intersection without switching filaments or getting stuck. (C) Probability that a cargo switched actin filaments. The error bars illustrate the standard error of the outcomes, which is no greater than approximately 0.3%. The lines connecting points are merely guides for the eye; they do not imply a specific functional relationship.

**Figure 3:**
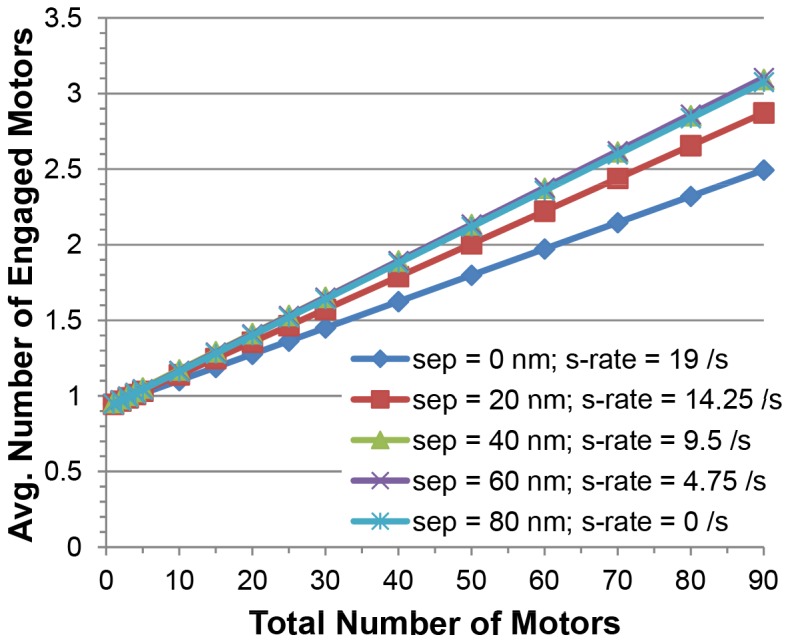
Average number of engaged motors that actively hauled a cargo versus the total number of motors that were on the cargo for different vertical separations between the two filaments. The lines represent linear regression fits to the results. Standard error of the average engaged motors is less than 0.1 and is not shown since it is smaller than the plot markers.

Figure 2 shows the probability that a cargo passed through an intersection, switched filaments, or fell off at or before the intersection, as a function of the total number of motors on the cargo for no vertical spacing between the filaments. Since the motors were randomly spread over the surface of the cargo, the average number of motors engaged in actively hauling the cargo along an actin filament was low relative to the total number of motors present [8], and increased linearly from 1 to about 2.6 as the total number of motors on the cargo increased from 1 to 90 motors for crossing filaments that touched (see Figure 3). As vertical separations between filaments increased, there was less competition between filaments for motor binding, so that the slope of this line increased slightly with increasing vertical separation; for separations between 40 and 80 nm, there were an average of about 3 actively engaged motors when there were 90 total motors on the cargo as shown in Figure 3.

From Figure 2, what happened to a cargo depended significantly on the arrangement of the filaments. If the cargo was moving along a filament and oriented such that it could ‘cross’ the intersection without ‘stepping over’ the second filament (“cargo starting from below” in Figure1C), the intersection had little effect, and the cargo usually (about two thirds of the time) continued to move along the filament it was on. This was approximately independent of the number of motors present on the cargo, though there was some (∼15–32%) chance of falling off at or before the intersection, with this probability decreasing slightly with an increase in the number of motors present, reflecting an increase in mean travel distance with an increased number of engaged motors. Occasionally, in about 10–20% of the runs, the cargo switched to the crossing filament since the intersecting filament was within reach of the motors on the cargo even though the cargo started below the first filament. Thermal fluctuations helped to occasionally bring the cargo closer to the crossing filament.

In contrast, if the cargo was on a filament and oriented such that it started from above (Figure1B), then the motor(s) had to ‘step over’ the second filament to continue on their way if the second filament crossed above the first with no vertical separation. Let us consider the case where the number of motors on the cargo was small. Here, the probability of stopping or falling off at an intersection was quite significant (about a third of the time). Occasionally, about 15–20% of the time (Figure2B), the motors on the cargo got through the intersection by stepping over, or onto and then over, the crossing filament. About half the time (Figure2C), the actively engaged motors switched the cargo to the crossing filament.

Figure 2 shows that as the number of motors on the cargo increased from 1 to about 10, the probability of going through the intersection increased slightly, while the probability of switching, stopping or falling off at an intersection decreased. The accompanying slight increase in the number of engaged motors (Figure3) helped the cargo to keep going on the original filament but there were not enough additional motors to promote switching. This is consistent with our original hypothesis that the more motors there were on the cargo, the more motors that would be actively hauling the cargo along a filament and hence, the harder it would be for another motor on the cargo to pull the cargo onto an intersecting filament because it would have to overcome several motors walking along the original filament.

However, as the number of motors increased above 10, the probability of these stopping events decreased, and concurrently the probability of the switching events increased slightly. We hypothesized that the initiator of both processes was the same. When more motors were present, these additional motors could now attach to the crossing filament that was blocking the cargo's forward advance. In this case, when the first motor detached, rather than the cargo diffusing away, these other motors were then able to move the cargo along the second filament, leading to a switching event. Notice, however, that Figure 2 shows that increasing the number of motors had only a modest effect on the switching probability, from about 45%–50% for a few motors to a about 65% for 90 motors. Thus, this relatively large change in the number of cargo-bound motors increased the percentage of cargos switching only by about 20%.

We note that these simulations were done assuming single myosin motors are able to switch directly between crossed filaments, with a 50% probability, as suggested by recent *in vitro* experiments [1]. While single motor switching is significant for low numbers of motors (less than 30 total motors on the cargo), the effect is less important when there are large numbers of motors on the cargo (see Figure S1 and the supplement S1where we compare results with and without this single-motor switching property).

Our previous experiments showed that in melanophores where there were only actin filaments and no microtubules, the probability of melanosomes switching at an intersection could be decreased from about 50% for 60 motors during the aggregation of melanosomes to 0 – 6% for 90 motors during the dispersion of melanosomes [2]. While we had hypothesized that much of this could likely be achieved by regulation of the number of active motors on the cargo, the simulations done here do not support such a hypothesis. Contrary to our experimental results, our simulations indicated that the switching probability *increased* slightly with increasing motor number. In addition, for no vertical separation between filaments, our results in Figure 2 suggested that filament-filament switching is much less sensitive to motor number than we imagined, and it appears that controlling the number of motors alone is likely not how cells regulate the switching probability of their cargos. Thus additional mechanism(s) are needed to regulate switching.

### Vertical separation between filaments strongly affected the filament-filament switching probability

For the cargo starting from above, we investigated how the separation between filaments was related to the probability of a cargo switching filaments, because the separation affects the rate at which an actively engaged motor switches as well as the ability of the cargo to dip underneath the crossing filament. The rate at which an actively engaged motor switches filaments must decrease for increasing vertical separations between filaments, eventually going to zero for vertical separations much larger than 74 nm, the measured distance between the two heads of a myosin V motor that is taking a step [7], since at that point the two heads cannot span the gap. In our simulations we incorporated this by decreasing the switching rate linearly from 19/s to 0 as the separation increased from 0 to 80 nm. In addition, the greater the vertical filament spacing, the easier it was for the cargo to dip underneath the crossing filament. Note that the ability of active but unengaged (passenger) motors on the cargo to attach to the crossing filament is not affected by the filament separation, as long as they can reach the crossing filament.

**Figure 4:**
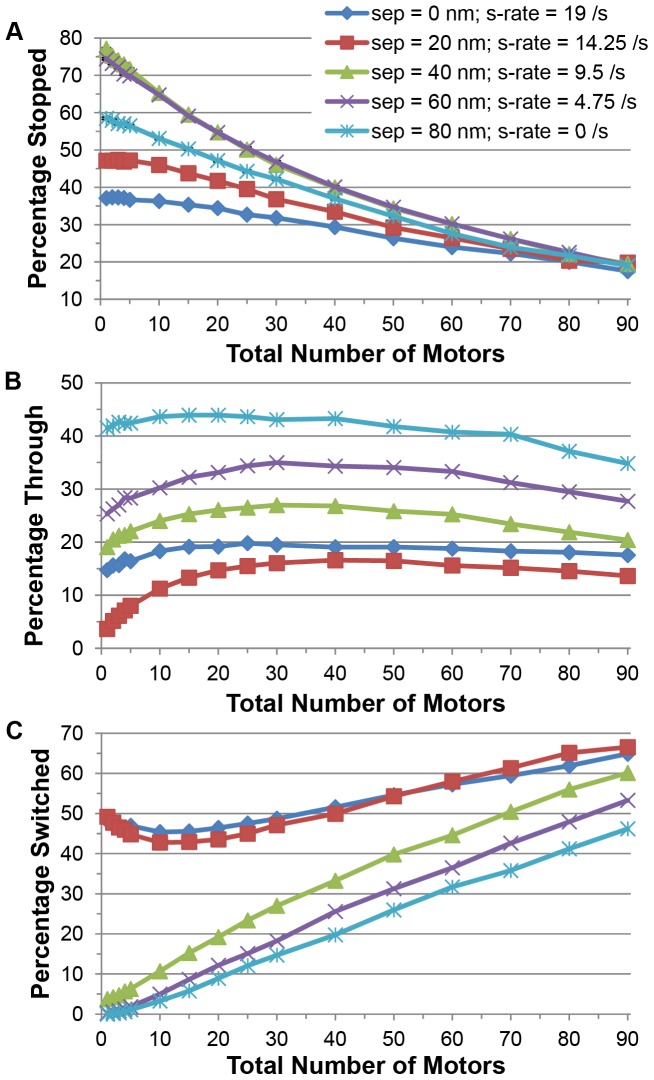
Probability of different outcomes for a cargo approaching an intersection versus the total number of motors on the cargo for vertical separations between filaments varying from 0 to 80 nm. The cargo started on top of the initial filament. The intersecting filament lay on top of the initial filament at an angle of 70 degrees. (A) Percentage of cargos that stopped at an intersection, i.e., percentage of cargos that detached at an intersection or before reaching the intersection. (B) Percentage of cargos that went through an intersection without switching filaments or getting stuck. (C) Percentage of cargos that switched actin filaments. The standard error of the outcomes is less than 0.3% and is not shown because the errors are smaller than the size of the plot markers. The lines connecting points are merely guides for the eye; they do not imply a specific functional relationship.

Figure 4 shows the percentage of cargos that stopped at (or before) the intersection, passed through the intersection, or switched filaments as a function of the total number of motors that were on the cargo for vertical separations δ ranging from 0 to 80 nm. As the number of motors increased, fewer cargos got stuck at the intersection (Figure 4A) and more cargos switched (Figure 4C). For a given separation, Figure 4B shows that the probability that a cargo passed through the intersection on the same filament did not change much with motor number. However, Figure 4B shows that there was a modest maximum between 10 and 50 motors for all separations as a function of the number of motors that were on the cargo. This was because as the number of motors increased from just a few motors, there was a greater chance that some of those motors attached to the crossing filament, followed by some motors stepping back onto the first filament which allowed the cargo to effectively step over the crossing filament and pass through the intersection. As the number of motors increased past the maximum in the probability to pass through the intersection (Figure 4B), the probability increased that some of the additional motors would attach to the crossing filament and switch the cargo onto this second filament. As a result, the percentage of switching increased and the probability of passing through the intersection decreased. Figure 4C shows that the switching probability increased only modestly with the total number of motors on the cargo for small filament separations (δ  =  0, 20 nm), but it increased dramatically for separations greater than or equal to 40 nm. For example, for δ  =  40 nm, the switching probability was only a few percent when there were a few motors but increased to about 60% for 90 motors.

**Figure 5:**
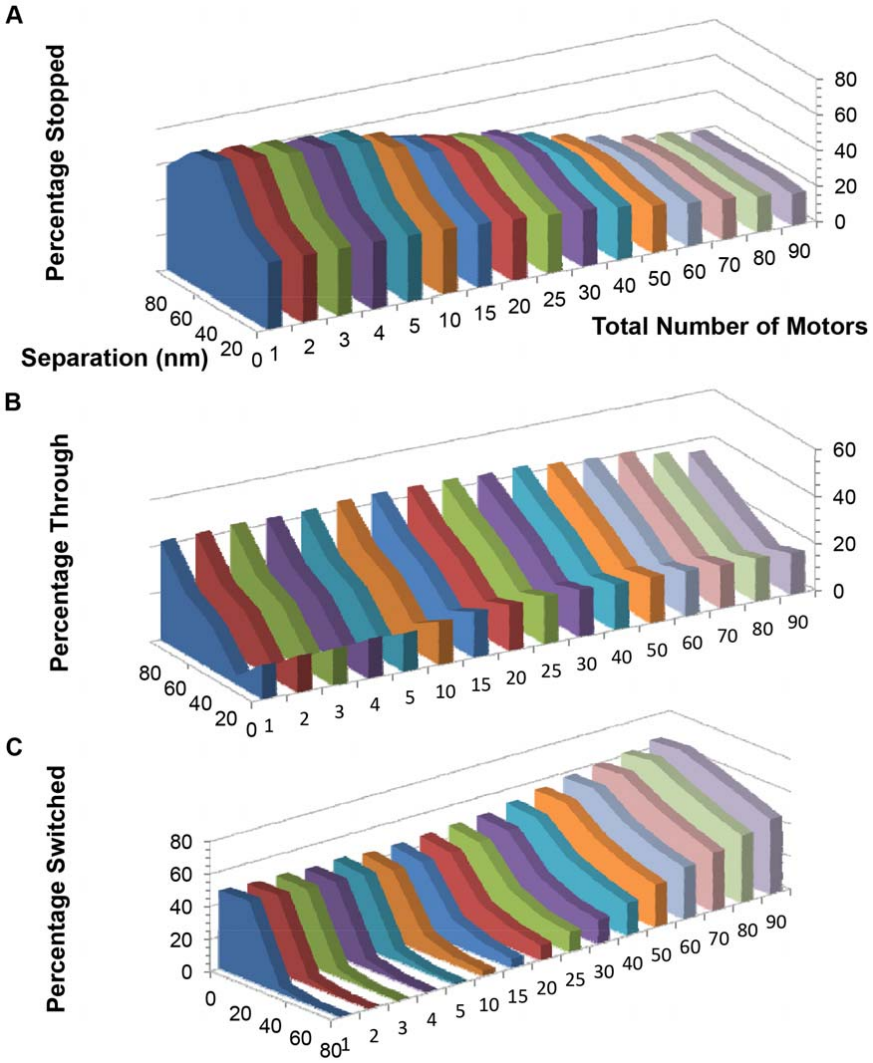
Percentage of different outcomes for a cargo that approached an intersection versus vertical separation between the two filaments and versus the total number of motors on the cargo. The cargo started on top of the initial filament. The intersecting filament lay on top of the initial filament at an angle of 70 degrees. (A) Percentage of cargos that stopped at an intersection, i.e., percentage of cargos that detached at an intersection or before reaching the intersection. (B) Percentage of cargos that went through an intersection without switching filaments or getting stuck. (C) Percentage of cargo that switched actin filaments. The standard error of the outcomes is no greater than 0.3% and is not shown.

To better understand the effect of vertical filament separation, we plot the probability of the various outcomes as a function of separation and the total number of motors on a cargo in Figure 5. In Figure 5A, the probability that a cargo got stopped at an intersection was between 15% and 50% for small separations (≤ 20 nm). Cargos with only a few motors were more likely (than cargos with many motors) to get stopped because there were not many other motors that could attach to the second filament. As the vertical separation increased, the chance of getting stopped did not change much with separation for cargos with 40 or more motors, but as separations increased from 0 nm up to about 40 nm, the stopping probability increased for cargos with only a modest number of motors. This increase was due to the decrease in the probability of an actively engaged motor switching, and was accompanied by the dramatic decrease in cargo switching seen in Figure 5C. As separations increased beyond 40 nm, the percentage of cargos that got stopped at the intersection decreased for cargos with only a modest number of motors because more cargos were able to pass through the intersection (see Figure 5B) by dipping underneath the second filament. Figure 5B shows that as the separation increased beyond 20 nm, it was easier for the cargo to dip underneath the crossing filament and pass through the intersection regardless of the total number of motors on the cargo. As the separation increased from 0 to 20 nm, Figure 5B shows that cargos with only a modest number of motors had increasing difficulty to pass through the intersection because it was too hard to step over the crossing filament, the cargo was too big to fit between the filaments, it was hard for it to dip underneath the crossing filament, and it was less likely that motors stepped up onto the second filament and then back onto the first filament because the rate of an actively engaged motor switching to the second filament decreased with increasing separation.

We see from Figure 5C that the switching probability decreased with increasing filament separation regardless of the total number of motors on the cargo as expected. This decrease was particularly abrupt for filament separations between 20 and 40 nm for cargos with less than about 40 motors. This decrease was due to the decreased switching rate for actively engaged motors, the increased ease for the cargo to continue through the intersection by dipping below the crossing filament (see Figure 5B), and the increased probability to stop at the intersection (see Figure 5A). For separations larger than 40 nm, the cargos with only a few motors had a very small probability of switching filaments since actively engaged motors had difficulty reaching the second filament and these cargos did not have many excess ‘passenger’ motors that could attach to the second filament. Cargos with greater numbers of motors were better equipped to switch filaments since they had other ‘passenger’ motors that could attach to and walk along the second filament once the engaged motors on the original filament detached.

## Discussion

Contrary to experiment where increasing the number of motors on the cargo decreased the probability of switching between filaments [2], our simulations found that the switching probability increased with the number of motors for a fixed filament separation. Part of the reason for this reflects a kinetic effect: if a motor binds to an intersecting filament, the cargo slows down and allows other motors to bind to the intersecting filament. Furthermore, even when there are a large number of motors on a cargo, only a few motors are engaged [8]. For example, in our previous simulations [8], a cargo with a diameter of half a micron and a total of 50 motors randomly attached to its surface, only has 5 actively engaged motors on average. Since only a few motors are attached to each filament at an intersection, the tug of war teams are roughly equally matched. In addition, the ability of a single engaged motor to switch filaments decreases the sensitivity of the switching probability to motor number, provided the intersecting filaments are close to each other or touching.

Our results imply that for small vertical filament separations (Figures 2, 4, and 5), the probability to switch filaments cannot be controlled effectively by simply adjusting the total number of motors on the cargo. However, as Figures 4 and 5 show, we do find evidence that switching can be controlled by adjusting ***both*** the total number of active motors on the cargo as well as the vertical separation between filaments. As Figure 5C shows, for cargos with only a modest number of active motors, the switching probability can be changed from about 50% to only a few percent by changing the separation between filaments from 20 nm (or less) to 40 nm (or more). Cargos with a large number of active motors (50 or more for a half micron diameter cargo) can still switch frequently with large filament separation, though less frequently than for small filament separation. Thus, a possible explanation for our previous experiments on melanosome switching is that the probability of switching at an intersection could be substantially tuned by regulating filament spacing. There exist many actin-crosslinking proteins [12], [13] that can produce different filament-filament spacings such as fimbrin and α-actinin [14]. Typical separations between actin filaments vary between 12 and 50 nm, consistent with the range of filament-filament spacing necessary to significantly alter switching probability. The extent to which this strategy – of modulating filament spacing to alter filament-to-filament switching of cargos – is employed in actual cells remains an exciting area for future exploration.

## Supporting Information

Figure S1 **Probability of different outcomes for a cargo approaching an intersection versus the total number of motors on the cargo with no vertical separation between filaments.** “Above” means that the cargo started on top of the initial filament. “Below” means that the cargo started on the bottom of the initial filament. The intersecting filament lay on top of the initial filament at an angle of 70 degrees. “No Switch” means that a single engaged motor cannot switch between filaments. The lines that are not designated “no switch” allow single engaged motors to switch between filaments at a rate of 19/sec. (A) Percentage of cargos that stopped at an intersection, i.e., percentage of cargos that detached at an intersection or before reaching the intersection. (B) Percentage of cargos that went through an intersection without switching filaments or getting stuck. (C) Percentage of cargos that switched actin filaments. The error in the outcomes, not shown in the figures, was no greater than about 5% probability in all cases.
(TIF)

Table S1 **Myosin V motor input parameters used in Monte Carlo simulation.**
(DOCX)

Table S2 **Input parameters used in Monte Carlo switching simulation.**
(DOCX)

Supplement S1 **This supplement contains a description of (1) our simulations of the transport of three dimensional cargo switching between actin filaments; and (2) our results for what happens when a cargo approaches intersecting filaments with and without single-motor switching.** A derivation of the standard error of switching outcomes is also provided.
(DOCX)
