# Supplementary figures and images for: Filament-Filament Switching Can Be Regulated by Separation Between Filaments Together with Cargo Motor Number
Source: PLoS One. 2013 Feb 14;8(2):e54298. doi: 10.1371/journal.pone.0054298 (PMC3573032; doi:10.1371/journal.pone.0054298)

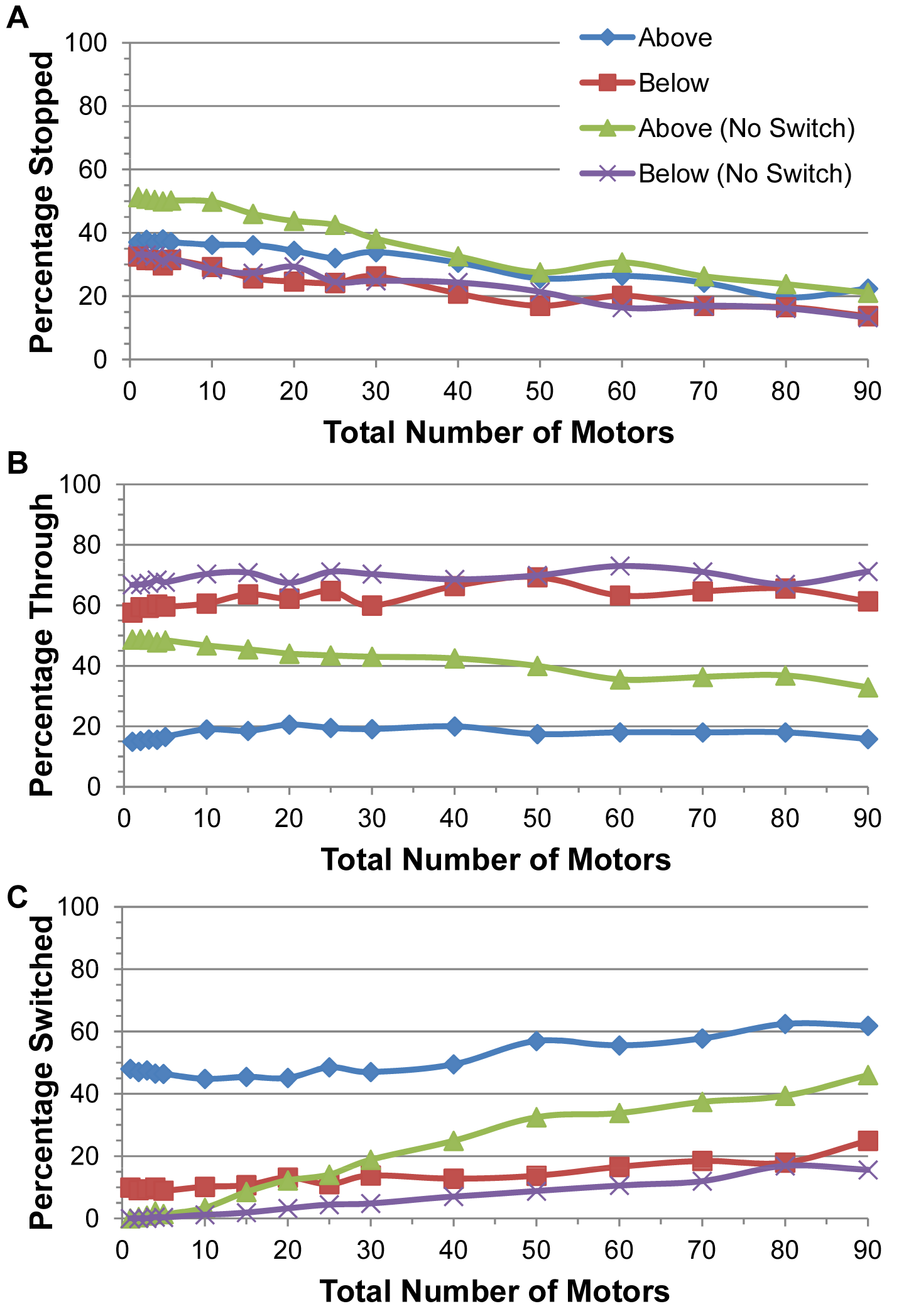

Supplement: Figure S1 — Probability of different outcomes for a cargo approaching an intersection versus the total number of motors on the cargo with no vertical separation between filaments. “Above” means that the cargo started on top of the initial filament. “Below” means that the cargo started on the bottom of the initial filament. The intersecting filament lay on top of the initial filament at an angle of 70 degrees. “No Switch” means that a single engaged motor cannot switch between filaments. The lines that are not designated “no switch” allow single engaged motors to switch between filaments at a rate of 19/sec. (A) Percentage of cargos that stopped at an intersection, i.e., percentage of cargos that detached at an intersection or before reaching the intersection. (B) Percentage of cargos that went through an intersection without switching filaments or getting stuck. (C) Percentage of cargos that switched actin filaments. The error in the outcomes, not shown in the figures, was no greater than about 5% probability in all cases. (TIF) [file pone.0054298.s001.tif]
