# Supplementary material for: Filament-Filament Switching Can Be Regulated by Separation Between Filaments Together with Cargo Motor Number
Source: PLoS One. 2013 Feb 14;8(2):e54298. doi: 10.1371/journal.pone.0054298 (PMC3573032; doi:10.1371/journal.pone.0054298)
Supplement: Table S1 — Myosin V motor input parameters used in Monte Carlo simulation. (DOCX) [file pone.0054298.s002.docx]

|  | | |
| --- | --- | --- |
| **Parameter** | **Value** | **Description** |
| [ATP] | 2000 μM | ATP concentration |
| d | 37 nm | discrete motor step length |
| k_on_ | 2 x 10^6^ M^-1^·sec^-1^ | ATP rate of binding |
| k_0off_ | 55 sec^-1^ | ATP rate of unbinding |
| k_cat_ | 105 sec^-1^ | ATP rate of hydrolysis |
| k_attach_ | 10 sec^-1^ | rate of attachment to actin |
| k_back-detach_ | 2 sec^-1^ | rate of attachment under load |
| d_1_ | 1.6 nm | distance associated with work of unbinding |
| δ_1_ | 1.3 nm | displacement associated with work of load |
| F_C_ | 13.5 pN | empirical constant with units of force |
| F_0_ | 1.8 pN | stall force |
| A | 107 | maximum motor steps before detachment |
| B | 0.029 μM | proportionality constant |
| T | 300 K | simulation temperature |
|  | | |
|  | | |
