# Supplementary material for: Filament-Filament Switching Can Be Regulated by Separation Between Filaments Together with Cargo Motor Number
Source: PLoS One. 2013 Feb 14;8(2):e54298. doi: 10.1371/journal.pone.0054298 (PMC3573032; doi:10.1371/journal.pone.0054298)
Supplement: Table S2 — Input parameters used in Monte Carlo switching simulation. (DOCX) [file pone.0054298.s003.docx]

|  | | | | |
| --- | --- | --- | --- | --- |
| **Parameter** | **Value** | **Description** |  |  |
| κ | 19 sec^-1^ | motor switching rate between actin filaments | |  |
| δ | 15 nm | vertical distance between actin filaments | |  |
| λ | 350 nm | travel distance to second actin filament | |  |
| φ | 70^0^ | Angle between intersecting actin filaments | |  |
| *η/η_water_* | 1 to 10 | fluid-to-water ratio of dynamic viscosity | |  |
| z_0_ | 15 nm | initial height of cargo above first actin filament | |  |
| R_AF_ | 3.5 nm | radius of either actin filament | |  |
| R | 250 nm | cargo radius | |  |
|  | 60 nm | motor linkage length | |  |
| k | 0.32 pN/nm | motor spring constant | |  |
| ϕ | 180^0^ | angle of motor distribution about cargo surface | |  |
|  |  |  | |  |
|  | | | |  |
