# Supplementary material for: Filament-Filament Switching Can Be Regulated by Separation Between Filaments Together with Cargo Motor Number
Source: PLoS One. 2013 Feb 14;8(2):e54298. doi: 10.1371/journal.pone.0054298 (PMC3573032; doi:10.1371/journal.pone.0054298)
Supplement: Supplement S1 — This supplement contains a description of (1) our simulations of the transport of three dimensional cargo switching between actin filaments; and (2) our results for what happens when a cargo approaches intersecting filaments with and without single-motor switching. A derivation of the standard error of switching outcomes is also provided. (DOCX) [file pone.0054298.s004.docx]

Supplement S1

In this supplement we describe (1) our simulations of the transport of three dimensional cargo switching between actin filaments; and (2) our results for what happens when a cargo approaches intersecting filaments with and without single-motor switching. A derivation of the standard error of switching outcomes is also provided.

Description of Simulation

We present the outline of the procedure for three-dimensional Monte Carlo simulation of a cargo switching between two identical intersecting cylindrical actin filaments. The simulation considers travel toward the positive end of one cylindrical actin filament, as driven by multiple single-myosin-V motors that step along the actin filament by repeated attachment and detachment, allowing for the probability of motors switching to the second actin filament. A motor’s stepping action is the Monte Carlo simulation of a mechanoenzyme displacement that involves a steady-state Michaelis-Menten process of ATP binding to the motor head, unbinding from the motor head, and hydrolysis [[1](#_ENREF_1),[2](#_ENREF_2)]. Reattachment is permitted to either actin filament, depending on the ability of the motor to reach; if the first actin filament is out of reach and the second is within reach, then attachment is made to the second actin filament. Additionally, a switching probability κ is imposed that permits an attached motor to switch from one actin filament to the other if the second actin filament is within reach. The actin filaments reside in two planes parallel to one another, separated by a distance δ, and intersecting at a distance λ from the start of simulated cargo travel. The cargo is taken to be a rigid sphere, with radius R and a density equal that of water at room temperature and atmospheric pressure. Cargo translational and rotational dynamics result from (i) forces and torques exerted by attached motors, (ii) random forces and torques exerted by the surrounding thermally fluctuating fluid medium, and (iii) an optionally applied external load acting on the center of mass of the cargo. The cargo interacts with the surrounding medium via Brownian motion, in accordance with the forms of Stokes law applicable to both a translating and rotating sphere immersed in a fluid with dynamic viscosity η.

Overviews of Monte Carlo simulation of cargo travel along filaments can be found in [[3](#_ENREF_3),[4](#_ENREF_4)]. The details of three-dimensional simulation of cargo travel along a single microtubule are presented in an earlier work [[4](#_ENREF_4)]. Here we describe the specifics of a Monte Carlo model of cargo switching between two actin filaments.

# Myosin V Motor Simulation

Our model of myosin-V motor displacement, in the form of discrete steps d, is a simulation of the steady-state enzyme reaction that describes ATP binding to the motor head, unbinding from the motor head, and hydrolysis. In this way, a motor travels along an actin filament via the motor head’s repeated attachment to and subsequent detachment from the actin filament. The rate of enzyme reaction can be denoted by a velocity V given by [[3](#_ENREF_3)]

$\begin{matrix} V=\frac{V_{\max}[ATP]}{[ATP]+K_{M}}; & V_{\max}=k_{cat}d\left[1-\left(\frac{F}{F_{0}}\right)^{2}\right]; & K_{M}=\frac{k_{cat}+k_{off}}{k_{on}} \end{matrix}$. (1)

Here, [ATP] is the concentration of ATP; K_M_ is the Michaelis-Menten constant; k_on_, k_off_, and k_cat_ are the rates of binding, unbinding, and hydrolysis of ATP, respectively; F is the magnitude of a load on the motor directed toward the negative end of the actin filament; and F_0_ is the motor stall force. The factor [1-(F/F_0_)^2^] in V_max_ interpolates between V_max_ = k_cat_d at no load and V_max_ =0 at the stalling force F_0_. This reproduces the experimentally measured force-velocity curve for kinesin [[3](#_ENREF_3),[5](#_ENREF_5)]. We are not aware of any experimental force velocity-curves for myosin V. Additionally, the rate of unbinding k_off_ depends on the magnitude of the load; it is modeled as

, (2)

where k_0off_ is the chemical rate of unbinding (in the absence of load), d_1_ is the distance over which the work of the load is done toward unbinding of ATP, T is the temperature, and k_B_ is the Boltzmann constant. The force F implicitly includes the effect of the thermally fluctuating cargo position and orientation due to the third law of motion.

A motor may exert a force and torque on the cargo, but only if its head is attached to the actin filament and its linkage is stretched. Attachment to the actin filament occurs at a rate defined by k_attach_. A motor head may detach from an actin filament during the simulation with a probability that depends on the current state of the motor. Specifically, if the motor resides in the state that occurs just before ATP binding, then the probability of detachment from the actin filament is P_detach1_ Δt, where Δt is a Monte Carlo time step, typically the order of 10^-5^ sec or less. If the motor resides in the state that occurs after ATP binding, then the probability of detachment from the actin filament is P_detach2_ Δt. The two detachment probabilities per unit time may be expressed as [[2](#_ENREF_2)]

$\begin{matrix} P_{\det tach1}=\frac{BP_{step}}{[ATP]} \\ P_{\det tach2}=\frac{P_{step}}{A}\exp\left(\frac{F_{C}}{F_{0}}\frac{F\delta_{l}}{k_{B}T}\right) \\ P_{step}=\left\{\begin{matrix} \frac{k_{cat}[ATP]}{[ATP]+K_{M}}\left[1-\left(\frac{F}{F_{0}}\right)^{2}\right] & ; & F<F_{0} \\ 0 & ; & F>F_{0} \end{matrix}\right. \end{matrix}$, (3)

where A is the maximum number of steps the motor head may take before detachment, B is a constant of proportionality, F_C_ is an empirical constant with units of force, F_o_ is the stall force, and δ_l_ is the displacement associated with the work done by the load force. The probability per unit time of taking a step of length d is given by P_step_. The rate k_back-detach_ is the rate of detachment under a load that is equal to or higher than the stall force F_0_. For Myosin V, we used a stall force F_0_ of 1.8 pN [[6](#_ENREF_6)].

The motor process simulated in our Monte Carlo calculations is also described in detail in [[2-4](#_ENREF_2)]. In particular, Erickson *et al.* [[4](#_ENREF_4)] discuss the theory of the three dimensional translational and rotational dynamics of cargo motion applied to the present simulation. In addition, motors are allowed to switch between actin filaments, even when initially attached to one actin filament. The particulars of the switching model are discussed in the next section. Table S1 summarizes the values of the input parameters that were used specifically in our mechanoenzyme simulation.

# Cargo Switching Simulation

In our Monte Carlo simulation a cargo is considered to reside on a particular actin filament if the majority of its motors that are attached to actin filaments are in fact attached to the actin filament in question. Thus, in our model, the simulation of cargo motor attachment to actin filaments determines cargo switching behavior.

Switching of motors between actin filaments can take place via two mechanisms. The first mechanism occurs when a motor detached from the first actin filament attaches to the second actin filament rather than reattaching to the first actin filament because the motor is within reach of the second but not the first. A motor is within reach of an actin filament if its unstretched linkage can make contact with the actin filament. The second mechanism occurs by introducing a model switching rate κ, which allows a motor attached to one actin filament to switch its attachment to the other actin filament with a probability κ Δt, provided the motor is within reach of the other actin filament. Again, Δt is a Monte Carlo time step of the order 10^-5^ sec or less. In this way, a cargo can switch residence from one actin filament to another if the majority of its motors attached to actin filaments are determined to have switched from one actin filament to the other.

When a cargo is sufficiently far from the intersection of two actin filaments, as governed by the length of a motor, the fraction of attached motors that forms this majority becomes unity. In fact, we begin our simulations with the cargo at a distance λ from the intersection of the first actin filament with a second, where λ is greater than the motor length. Thus, at the start of simulation, the cargo is known to reside on the first actin filament with certainty. At the end of a cargo simulation, when the motors of the cargo have detached from all actin filaments, such that the cargo is detached, we record the actin filament to which the majority of motors were attached just prior to cargo detachment, as well as the final location of the cargo. This defines the actin filament to which the cargo was attached just prior to its end of travel. The results, compiled from many repeated simulations, fall into three basic outcomes: (i) stop: the cargo detached from the first actin filament at or prior to the intersection of the two actin filaments, indicating it was unable to move past the actin filament intersection; (ii) through: the cargo detached from the first actin filament beyond the intersection, indicating it proceeded past the intersection without switching to the second actin filament; and (iii) switch: the cargo detached from the second actin filament beyond the intersection, indicating it proceeded past the intersection after having switched to the second actin filament.

Figure 1A in the paper illustrates two identical cylindrical actin filaments of infinite extent and radius R_AF_ over which a spherical cargo of radius R is permitted to travel. In this simulation motors are randomly attached over the full surface of the spherical cargo. The two actin filaments lie in parallel planes with respective directions of travel subtended by an angle φ, as illustrated in Figure 1A. At the intersection the surfaces of the two actin filaments are at a minimum distance of δ, taken to be smaller than the cargo radius. Simulations were performed for two basic initial conditions, one with the cargo starting out from the top surface of the first actin filament, at a distance λ from the intersection, as depicted in Figure 1B in the paper, and the other with the cargo starting out from the bottom surface of the first actin filament, also at a distance of λ from the intersection, as depicted in Figure 1C. In this way, a comparison was made between the switching outcomes of the two different starting conditions, top and bottom.

Table S2 shows the input values used for the simulation parameters we have discussed. In the next section we describe the Monte Carlo simulation algorithm in greater detail.

As a final comment we note that the model parameters k_attach_ = 10 /sec, F_C_ = 13.5 pN, and κ = 19 /sec were chosen to fit the stop, through, and switch outcomes of Figure 1A that were observed experimentally in [[7](#_ENREF_7)]. The parameters were independently adjusted until simulation outcomes matched experimental observations. Specifically, the values of k_attach_ = 10 /sec, F_C_ = 13.5 pN, and κ = 19 /sec were found to fit uniquely the 37% terminated, 15% stepped-over, and 48% switched frequencies of myosin Va molecules at actin filament intersections that were observed in the experiments of [[7](#_ENREF_7)].

# Monte Carlo Switching Simulation Algorithm

Our Monte Carlo switching simulation considers the travel along an actin filament by a three-dimensional, spherical cargo immersed in a fluid, subject to Stokes’ law. The cargo travels along the actin filament toward the intersection with a second actin filament, as depicted in Figure 1 in the paper. The simulation is carried out by employing the three distinct stochastic processes of mechanoenzyme motors, translational Brownian motion of the cargo, and rotational Brownian motion of the cargo. These processes are described in greater detail in [[4](#_ENREF_4)]. Each of these processes influences the propagation of the cargo toward the actin filament intersection. In addition, we allow an engaged motor to switch filaments in the vicinity of an intersection at a rate of 19/sec. Even without this provision, an engaged motor can switch filaments by detaching from the actin filament, and then attaching to the other actin filament. This happens when the cargo is tethered to the filament(s) by other motors.

Below we outline the steps of the algorithm:

*Simulation Start:*

1. For a given external load, N motors are randomly attached to the cargo within the region of the specified cluster angle. In all our simulations, we made the cluster angle 180-degrees, which means motors could attach anywhere on the surface of the cargo. The cluster angle is the polar angle measured from the z-axis. Motors can attach anywhere within a cone defined by the cluster angle and a 360 degree azimuthal angle about the z-axis measured from the x-axis.

   *New Initial Boundary Condition:*
2. With the cargo suspended above the first actin filament at a distance z_0_, as in Figure 1B, or below, as in Figure 1C, an attempt is made to attach each of the N motors to the actin filament. A motor cannot attach if its linkage passes through the volume of the cargo. If no motors can be attached, then the cargo is rotated so that one of the attachment points of a motor to the cargo is directly over (Fig. 1B) or under (Fig. 1C) the actin filament. As long as the relaxed length of this motor’s linkage is at least z_0_, then at least one motor is guaranteed to attach to the actin filament.

   *New Cargo Travel Scenario:*
3. With the initial boundary condition established, such that a subset of N motors is now attached to the actin filament, a travel scenario is constructed by partitioning the passage of time into equal intervals Δt, starting from the initial time.

   *New Scenario Time Step:*
4. At a given interval of time, the following steps are taken:
   1. The instantaneous net force and torque acting on the cargo is computed.
   2. Each of the N motors is allowed to follow the process of stepping toward the plus end of the actin filament, which is an approach to the intersection with a second actin filament. The process followed by a motor may be described as follows:
      1. If the motor is detached from an actin filament, then a test is made to attach the motor, according to the probability of attaching $k_{attach}\Delta t$. The test is made by drawing a random variate from the uniform distribution, on the interval [0,1], and comparing it to the stated probability. If the test succeeds, which means the variate is less than the probability, then the motor is attached to the first actin filament to which it can reach via an unstretched linkage length. If no actin filament is within reach, then the test fails.
      2. If the motor is attached to a actin filament, then the following tests may occur:
         1. A test is made to see if the motor can switch to an adjacent actin filament provided a non-negative switching rate κ is specified:
            1. If this is the case then a random variate is drawn on the interval [0,1] and compared to the probability $\kappa\Delta t$. If the variate is less than $\kappa\Delta t$, then an attempt is made to attach the motor to an actin filament other than the one to which it is currently attached. If the motor can reach another actin filament with an unstretched linkage, then it is attached to this actin filament instead.
            2. If the switching rate κ is zero or the motor fails to attach to another actin filament, then the procedure continues to the next test, below.
         2. A test is made to see if the motor can detach from the actin filament according to either of the probabilities $P_{\det tach\text{ }1}\Delta t$, $P_{\det tach\text{ }2}\Delta t$, as computed via (3). If the test succeeds for either mode of detachment, then the motor is detached from the actin filament, which completes the process.
         3. If the test fails, then activating or deactivating the motor, i.e., ATP binding or unbinding, is implemented:
            1. If ATP is bound, then a test is made to determine if ATP is released using the probability $k_{off}\Delta t$, via (2). If the test succeeds, then ATP is released, which completes the process.
            2. If the head has no ATP, then a test is made to determine if ATP will bind using the probability $k_{on}\left[ATP\right]\Delta t$. If the test fails, such that there is still no ATP bound, then an additional test is performed to determine if the motor has stalled, i.e., whether the load F exceeds the stall force F_0_. If the load exceeds the stall force, then a random variate drawn on the interval [0,1) is tested against the probability $k_{back-\det ach}\Delta t$ to simulate the possibility of the motor detaching from the actin filament under a stalling load.
         4. To reach this step, the ATP must be bound to the head, either because ATP attached before the process started or because it attached during the process of step 2, above. Since the motor is active, a test of ATP hydrolysis can be made using the probability of $k_{cat}\Delta t$. If this hydrolysis test succeeds, then ATP is converted to ADP (which is released) and a test of the motor taking a step can be performed. If the test fails, then an additional test for detachment is made. The detachment test is the same as described in 2b, above. If this additional test succeeds, then the motor is detached from the actin filament, which completes the process.
         5. If the process continues to this point, then a test is made to see if the motor may take a step along the actin filament. The test can be made against the probability $\varepsilon$, where $\varepsilon=1-\left(F/F_{0}\right)^{2}$ is the efficiency function. If the test succeeds, then the motor may make a step provided its linkage does not pass through the volume of the cargo. If the test fails, then a detachment test like that of 2b is performed.
   3. The center of mass of the cargo is translated using the net force computed in step a. This involves drawing a normally distributed variate corresponding to each of the three Cartesian coordinates. The new position is rejected if it means that the cargo or motor (linkages) will pass through solid objects, i.e., cargo or actin filaments.
   4. The cargo is rotated using the net torque computed in step (a) and instantaneous Euler angles. This also involves drawing a normally distributed variate corresponding to each of three Euler angles. The new position is rejected if it means that the cargo or motor (linkages) will pass through solid objects, i.e., cargo or actin filaments.
5. Step 4 is repeated until all N motors are detached from actin filaments, at which point the travel scenario has ended. The final time step determines how far the cargo has traveled, i.e., the run length of the cargo for this scenario. The actin filament from which the cargo detaches and the position of the cargo at the point of detachment determines the switching outcome. Switching outcomes are depicted in Figure 1A.
6. Step 1 is repeated for either of the two starting orientations, top and bottom, as depicted in Figure 1B and 1C.

**Effect of Allowing a Single Engaged Motor to Switch Filaments**

Engaged motors actively hauling a cargo along a filament can switch to an intersecting filament by detaching from the initial filament and then attaching to the crossing filament, as long as at least one other motor tethers the cargo to a filament. In addition, an engaged myosin-V motor can simply switch its attachment from one filament to the other intersecting filament while tethering a cargo [[7](#_ENREF_7)]—a process we refer to as single-motor switching. In our simulations we use a single-motor switching rate of 19/s in order to achieve agreement with the experimental values of [[7](#_ENREF_7)].

To determine the effect of this latter switching mechanism, we compared the results with and without single-motor switching. The simulations shown here were done somewhat differently from the paper. In the present case, 25 different random attachments of all the motors to the full spherical surface of the cargo were considered. For each of these 25 different initial stochastic boundary conditions, 1000 simulations were run wherein the motors randomly attached to the first filament and the cargo proceeded to walk down the first filament toward the intersection with the second filament. This resulted in 25 x 1000 = 25,000 simulated outcomes in which the cargo either (i) stopped at or before the intersection, (ii) passed through the intersection without switching to the second filament, or (iii) switched to the second filament. Figure S1 shows the resulting probability of each of the three outcomes as a function of the total number of motors attached to the cargo. Simulations were performed with the cargo starting out from both above and below the first filament, with no separation between the intersecting filaments. Also, simulations were performed wherein the 19/s single-motor switching rate was turned off. The errors, not shown in the figure, are no greater than about 5% probability in all cases. The present simulations differ from that of the paper in that those of the paper involve 25,000 different initial stochastic boundary conditions rather than 25, such that each of the 25,000 cargo traversals down the first filament is completely independent of the other, resulting in a much smaller error in the reported outcomes.

When the cargo started on the bottom of the initial filament, the results did not depend much on whether there was single motor switching. However, when the cargo started on top of the initial filament, single-motor switching had a significant effect on the percentage of cargos that went through the intersection and that switched. As Figure S1B shows, turning off single-motor switching enhanced the probability of passing through the intersection by about 20% (from about 20% to 40%). This makes sense since the cargo switching was diminished without single-motor switching. Figure S1C shows that if there were fewer than 30 motors on the cargo, then the percentage of cargos that switched filaments was about 50% if single-motor switching was allowed compared to virtually no switching (less than 5%) if there was no single-motor switching. This means that for a small number of motors, almost all of the switching was due to single-motor switching.

The other switching mechanism, wherein the engaged motor detached from the filament and then switched by attaching to the other filament, was not so effective when there was a small number of motors on the cargo because it was likely that no other motors were attached to filaments. Thus, in this instance, once the engaged motor detached, the cargo would diffuse away from the filament and switching did not occur. As the total number of motors on the cargo increased, this type of switching became more pronounced. Moreover, when combined with the single-motor switching mechanism, the overall cargo switching probability increased by about 15-20% (from about 40% to 60%) for cargos with 80 to 90 motors, as evidenced by the blue and green curves in Figure S1C.

# Derivation of the Standard Error of Switching Outcomes

The Monte Carlo simulations described in the paper consisted of three types of outcomes that we can summarize as: (1) the cargo stopped at the intersection between filaments, (2) the cargo passed through the intersection without switching to the second filament, and (3) the cargo switched to the second filament. Each of these three types of outcomes is associated with a probability that can be labeled as p_1_, p_2_, and p_3_, respectively. Each of the three probabilities is a real number that can range in value between 0 and 1, with the constraint that p_1_ + p_2_ + p_3_ = 1.

Consider any one of these three types of outcomes, say the i-th outcome, whose probability may be expressed as p_i_. Suppose N Monte Carlo simulation scenarios are run such that N outcomes $x_{1}^{(i)},x_{2}^{(i)},\ldots,x_{N}^{(i)}$ are recorded from the perspective of the i-th type of outcome. Here the j-th outcome is either $x_{j}^{(i)}=1$ upon success, i.e., the i-th type of outcome is observed, or $x_{j}^{(i)}=0$ upon failure, i.e., one of the other two types of outcomes is observed.

If the N scenarios are independent of one another than the likelihood of obtaining such a set of simulation results may be expressed as

$\mathcal{L}_{N}\left( p_{i} \right)={p_{i}}^{x_{1}^{(i)}}\left( 1-p_{i} \right)^{1-x_{1}^{(i)}}\ldots{p_{i}}^{x_{N}^{(i)}}\left( 1-p_{i} \right)^{1-x_{N}^{(i)}}={p_{i}}^{\sum_{j=1}^{N} x_{j}^{(i)}}\left( 1-p_{i} \right)^{N-\sum_{j=1}^{N} x_{j}^{(i)}}$. (4)

The most likely value for $p_{i}$, call it $p_{i}^{*}$, is the maximum of the function of equation (4). Hence, $p_{i}^{*}$ satisfies

$\left. \frac{d}{dp_{i}}\mathcal{L}_{N}\left( p_{i} \right) \right|_{p_{i}=p_{i}^{*}}=\left. \mathcal{L}_{N}\left( p_{i}^{*} \right)\frac{d}{dp_{i}}\log\mathcal{L}_{N}\left( p_{i} \right) \right|_{p_{i}=p_{i}^{*}}=0$. (5)

Applying equation (4) to (5), and assuming $\mathcal{L}_{N}\left( p_{i}^{*} \right)\neq0$, we find

$p_{i}^{*}=\frac{1}{N}\sum_{j=1}^{N} x_{j}^{(i)}$, (6)

which, in this particular case, is the same as the sample mean.

Noting the above result, the standard error may be expressed as the square root of

${{\Delta p}_{i}}^{2}=\frac{\int_{0}^{1} {\left( p_{i}-p_{i}^{*} \right)^{2}\mathcal{L}}_{N}\left( p_{i} \right)dp_{i}}{\int_{0}^{1} \mathcal{L}_{N}\left( p_{i} \right)dp_{i}}$. (7)

Now, for $N\gg1$, the likelihood function $\mathcal{L}_{N}\left( p_{i} \right)$ of equation (4) is steeply peaked about the value $p_{i}=p_{i}^{*}$. If we expand the logarithm $\log\mathcal{L}_{N}\left( p_{i} \right)$ in a Taylor series, in powers of $p_{i}-p_{i}^{*}$, noting that the first derivative of the logarithm is zero by construction, then

$\mathcal{L}_{N}\left( p_{i} \right)\cong\mathcal{L}_{N}\left( p_{i}^{*} \right)\exp\left\{ \frac{1}{2}\left( p_{i}-p_{i}^{*} \right)^{2}\left. \frac{d^{2}}{d{p_{i}}^{2}}\log\mathcal{L}_{N}\left( p_{i} \right) \right|_{p_{i}=p_{i}^{*}} \right\}$. (8)

Applying equation (8) to the numerator of (7), and integrating by parts, we obtain

$\int_{0}^{1} {\left( p_{i}-p_{i}^{*} \right)^{2}\mathcal{L}}_{N}\left( p_{i} \right)dp_{i}\cong-\left[ \left. \frac{d^{2}}{d{p_{i}}^{2}}\log\mathcal{L}_{N}\left( p_{i} \right) \right|_{p_{i}=p_{i}^{*}} \right]^{-1}\int_{0}^{1} \mathcal{L}_{N}\left( p_{i} \right)dp_{i}; N\gg1$. (9)

Thus, equation (7) may be approximated as

${{\Delta p}_{i}}^{2}\cong-\left[ \left. \frac{d^{2}}{d{p_{i}}^{2}}\log\mathcal{L}_{N}\left( p_{i} \right) \right|_{p_{i}=p_{i}^{*}} \right]^{-1}; N\gg1$. (10)

If we apply equation (4) to (10), and make use of (6), the standard error is approximately

${\Delta p}_{i}\cong\sqrt{\frac{p_{i}^{*}\left( 1-p_{i}^{*} \right)}{N}}; N\gg1$. (11)

1. Schnitzer MJ, Visscher K, Block SM (2000) Force production by single kinesin motors. Nat Cell Biol 2: 718-723.

2. Kunwar A, Vershinin M, Xu J, Gross SP (2008) Stepping, strain gating, and an unexpected force-velocity curve for multiple-motor-based transport. Curr Biol 18: 1173-1183.

3. Singh MP, Mallik R, Gross SP, Yu CC (2005) Monte Carlo modeling of single-molecule cytoplasmic dynein. Proc Natl Acad Sci U S A 102: 12059-12064.

4. Erickson RP, Jia Z, Gross SP, Yu CC (2011) How molecular motors are arranged on a cargo is important for vesicular transport. PLoS Comput Biol 7: e1002032.

5. Visscher K, Schnitzer MJ, Block SM (1999) Single kinesin molecules studied with a molecular force clamp. Nature 400: 184-189.

6. Schroeder HW, 3rd, Mitchell C, Shuman H, Holzbaur EL, Goldman YE (2010) Motor number controls cargo switching at actin-microtubule intersections in vitro. Curr Biol 20: 687-696.

7. Ali MY, Krementsova EB, Kennedy GG, Mahaffy R, Pollard TD, et al. (2007) Myosin Va maneuvers through actin intersections and diffuses along microtubules. Proc Natl Acad Sci U S A 104: 4332-4336.
